# FC-YOLO: a fast inference backbone and lightweight attention mechanism-enhanced YOLO for detecting gastric adenocarcinoma in pathological image

**DOI:** 10.3389/fonc.2025.1657159

**Published:** 2025-09-29

**Authors:** Hengtong Zhang, Jianxin Jia, Wenlian Zhang, Rigui Yi, Xusheng Yan, Wenyue Sun, Xinxin Wang, Yunfei Gao

**Affiliations:** ^1^ Basic and Forensic Medicine, Baotou Medical College, Inner Mongolia, Baotou, China; ^2^ Key Laboratory of Human Anatomy at Universities of Inner Mongolia Autonomous Region, Inner Mongolia, Baotou, China; ^3^ School of Computer Science and Technology, Baotou Medical College, Inner Mongolia, Baotou, China; ^4^ School of Medical Technology and Anesthesia, Baotou Medical College, Inner Mongolia, Baotou, China

**Keywords:** gastric cancer, pathological images, prediction, deep learning, target detection

## Abstract

**Background:**

Gastric adenocarcinoma (GAC) is a leading cause of cancer-related mortality, but its histopathological diagnosis is challenged by image complexity and a shortage of pathologists. While deep learning models show promise, many are computationally demanding and lack the fine-grained feature extraction necessary for effective GAC detection.

**Methods:**

We propose FC-YOLO, an optimized object detection framework for GAC histopathological image analysis. Based on the YOLOv11s architecture, FC-YOLO incorporates a FasterNet backbone for efficient multi-scale feature extraction, a lightweight Mixed Local-Channel Attention (MLCA) mechanism for feature recalibration, and Content-Aware ReAssembly of FEatures (CARAFE) for enhanced upsampling. The model was evaluated on a public dataset comprising 1,855 images and on a separate, independent clinical dataset consisting of 2,500 pathological images of gastric adenocarcinoma.

**Results:**

On the public dataset, FC-YOLO achieved a mean Average Precision (mAP) of 82.8%, outperforming the baseline YOLOv11s by 2.6%, while maintaining a high inference speed of 131.56 FPS. On the independent clinical dataset, the model achieved an mAP of 85.7%, demonstrating strong generalization capabilities.

**Conclusion:**

The lightweight and efficient design of FC-YOLO enables superior performance at a low computational cost. It represents a promising tool to assist pathologists by enhancing diagnostic accuracy and efficiency, particularly in resource-limited settings.

## 1 Introduction

Although the global incidence rate of gastric cancer has declined over the past few decades, it remains the fifth most common cancer type worldwide and the fourth leading cause of cancer-related mortality (1). In terms of pathological classification, over 95% of gastric cancers are adenocarcinomas (2). Early detection, accurate diagnosis, and timely surgical intervention are critical to reducing gastric cancer mortality. Histopathological diagnosis serves as the gold standard for confirming gastric cancer, and its outcomes significantly influence treatment planning, underscoring the necessity of robust and efficient pathological diagnostics. However, there is a severe shortage of pathologists globally, including in China (3). Additionally, due to the complexity of pathological images, the analysis and diagnostic process is inherently challenging and time-consuming, which may compromise diagnostic accuracy. As caseloads increase, pathologists face heightened workloads and occupational overload, further exacerbating diagnostic inaccuracies—a problem particularly pronounced in remote and underdeveloped regions. Therefore, the development of computer-aided diagnosis (CAD) tools capable of assisting pathologists and enhancing both the efficiency and accuracy of diagnosis holds significant clinical importance.

Recent years have witnessed remarkable advancements in deep learning for computer-aided diagnosis of medical images, particularly in gastric lesion detection and diagnosis systems. Compared with traditional machine learning methods such as random forests and support vector machines, deep learning demonstrates superior capability in capturing discriminative features from medical images with enhanced flexibility and diagnostic accuracy. In routine clinical pathological practice, histopathological examination of specimens, typically through hematoxylin and eosin (H&E) stained slides, is conventionally conducted under optical microscopy. Notably, deep convolutional neural networks (CNNs) have emerged as pivotal tools in computer vision and medical image analysis. The application of CNNs in digital pathological image analysis for gastric disease classification has garnered significant research attention. Models based on the DeepLabv3 architecture, as employed by Song et al. (4). and Lan et al. (5)., have demonstrated high accuracy in gastric cancer detection. However, these models exhibit fluctuations in accuracy under specific scenarios. Furthermore, their inference times are often prolonged, which may impede diagnostic efficiency in clinical settings that demand the rapid processing of a large volume of samples, thereby highlighting a need for improvement in their real-time performance. Huang et al. (6). designed Gastro-MIL, a CNN-based model for the accurate diagnosis of gastric cancer directly from digital H&E-stained images. While its diagnostic performance was reported to surpass that of junior pathologists, the model’s processing pipeline is notably complex. It requires extensive partitioning of input images into tiles and relies on a multi-stage network architecture, which likely contributes to increased computational overhead and prolonged inference times. Kather et al. (7). trained a ResNet18 deep learning model to detect gastric cancer and predict Microsatellite Instability (MSI) in histological slides. Although their model is characterized by a low parameter count, it demonstrates notable deficiencies in accuracy, achieving an Area Under the Curve (AUC) of only 0.69 in a predominantly Asian cancer cohort, which suggests limited generalization capability across different ethnic populations. Zhang et al. (8). proposed a model named VENet, which accurately segments glands in pathological images. However, the model employs a complex network architecture and loss function, incorporating a multi-scale training strategy and an iterative optimization process, which increases computational complexity and results in high deployment and training costs. In a related study, Shi et al. (9). introduced GCLDNet, a deep learning model that, despite its excellent performance on the BOT dataset, may suffer from reduced detection accuracy for minute lesions. Furthermore, the model lacks validation on clinically acquired samples, and its large parameter count restricts its application on low-resource devices. Liang et al. (10). employed a weakly supervised learning approach for the subcellular-level segmentation of gastric adenocarcinoma, achieving an accuracy of 0.9109 on the BOT dataset. Although this method reduces the annotation burden for pathologists, its recognition capability in specific scenarios is insufficient, making it prone to misdiagnosis. Additionally, the model requires a lengthy training period, and its inference time is highly sensitive to parameter settings. Ning et al. (11). synergistically utilized U-Net and QuPath for diagnosing gastric adenocarcinoma; however, the model’s generalization ability is constrained by a small sample size, and it similarly suffers from a complex processing workflow that prolongs inference time. Fu et al. (12) integrated Transformer and CNN architectures for the multi-class classification of gastric cancer pathology images, which enhanced model accuracy but concurrently increased computational overhead. Furthermore, the windowed attention mechanism and feature fusion process inherent to the Swin Transformer component increased per-sample processing time, leading to extended inference durations. Meanwhile, the application of the YOLOv4 model by Tung et al. (13) for detecting gastric adenocarcinoma, which lacked specific optimizations for histopathological features, was hampered by issues such as prolonged inference times and poor generalization capability. In a more recent study, Ma et al. (14) proposed an ensemble deep learning framework that fused VGG16, ResNet50, and MobileNetV2. This framework demonstrated exceptional performance in gastric cancer classification, surpassing standalone models. However, the fusion of three models resulted in high computational complexity and increased per-image processing time, posing challenges for real-time clinical applications and deployment on edge devices. Lomans et al. (15) trained an nnU-Net to assist in the pathological diagnosis of hereditary diffuse gastric cancer. Although its performance in lesion size estimation and cell type quantification approached that of pathologists, it exhibited insufficient adaptability to the complex tumor microenvironment. Moreover, its reliance on large-scale, meticulously annotated data restricts its broader adoption in resource-constrained medical institutions.

In summary, mainstream deep learning models for the detection of gastric adenocarcinoma in pathological images continue to face several critical challenges. High-accuracy models are often characterized by high computational complexity and prolonged inference times, which constrains their practical deployment in resource-limited hospitals and impedes their ability to meet the real-time requirements of clinical workflows. Conversely, lightweight models typically lack the requisite accuracy for reliable diagnosis. To address these limitations, we propose the FC-YOLO model to establish a deep learning framework capable of concurrently achieving high accuracy, rapid inference, and a lightweight design, tailored to the unique challenges of gastric adenocarcinoma pathology detection. This model incorporates three key modifications to the YOLOv11s baseline: first, the backbone network is replaced with FasterNet to more efficiently extract multi-scale features while enhancing inference speed; second, a lightweight MLCA attention mechanism is introduced to bolster the model’s focus on critical diagnostic regions; and finally, the CARAFE upsampling operator is employed for a more precise restoration of lesion boundary details.

The contributions of this paper are as follows:

1. This study proposes an enhanced object detection framework for pathological images based on YOLOv11 architecture. We systematically integrate three key components: the FasterNet backbone network, MLCA attention mechanism, and CARAFE upsampling operator into the baseline model. This multi-component integration strategy significantly enhances detection accuracy while maintaining computational efficiency, offering a robust solution for target detection tasks in histopathological image analysis.
2. Our model achieves an mAP of 82.8% on the BOT gastric adenocarcinoma dataset, demonstrating a 2.6% performance enhancement compared to the baseline YOLOv11s architecture. This advancement establishes a novel framework for pathological image detection in gastric adenocarcinoma diagnostics, addressing critical challenges in clinical histopathology analysis.
3.Extensive comparative and ablation experiments have comprehensively validated the effectiveness of our model in detecting gastric adenocarcinoma within pathological images. This study presents an innovative modification of YOLOv11s for this purpose, and its findings are anticipated to be of considerable value to future researchers in the field of object detection who are focused on the identification and prediction of tumors in pathological imagery.

## 2 Proposed method

### 2.1 Pathological image recognition model based on the YOLO network

The YOLO (You Only Look Once) framework represents a breakthrough in object detection neural networks, capable of simultaneous object localization and classification through a unified network architecture. Unlike conventional region proposal-based methods, YOLO demonstrates superior processing speed by employing an end-to-end detection paradigm. Built upon convolutional neural networks (CNNs), this framework processes entire images in a single forward pass rather than analyzing multiple image patches sequentially. This unified approach eliminates redundant computations inherent in sliding window techniques, enabling real-time performance while maintaining detection accuracy (16). Through continuous architectural evolution, multiple YOLO variants have been developed to enhance detection capabilities. In this study, we adopt YOLOv11s as our foundational framework, specifically optimized for histopathological image analysis tasks. Our rationale for selecting YOLOv11s is rooted in its integration of several state-of-the-art architectural optimizations. In comparison to the well-established YOLOv8 model, YOLOv11 introduces the C3K2 module. This module optimizes information propagation throughout the network by partitioning the feature map and applying a series of smaller kernels, which enhances feature representation using fewer parameters and at a lower computational cost than the C2f module in YOLOv8. Furthermore, its innovative C2PSA module refines the model’s capacity for selective attention to regions of interest by applying spatial attention to the extracted features, all while maintaining a judicious balance between computational cost and detection accuracy. This architectural design affords YOLOv11 an advantage over YOLOv8 and its predecessors in scenarios requiring the precise detection of fine-grained object details. Consequently, this highly favorable performance-to-cost profile establishes it as an ideal starting point for our task of detecting gastric adenocarcinoma in pathological images, facilitating the integration and validation of our novel optimization modules without being encumbered by the overhead of an excessively large base model.

### 2.2 The architecture of the FasterNet network

**Figure 1:**
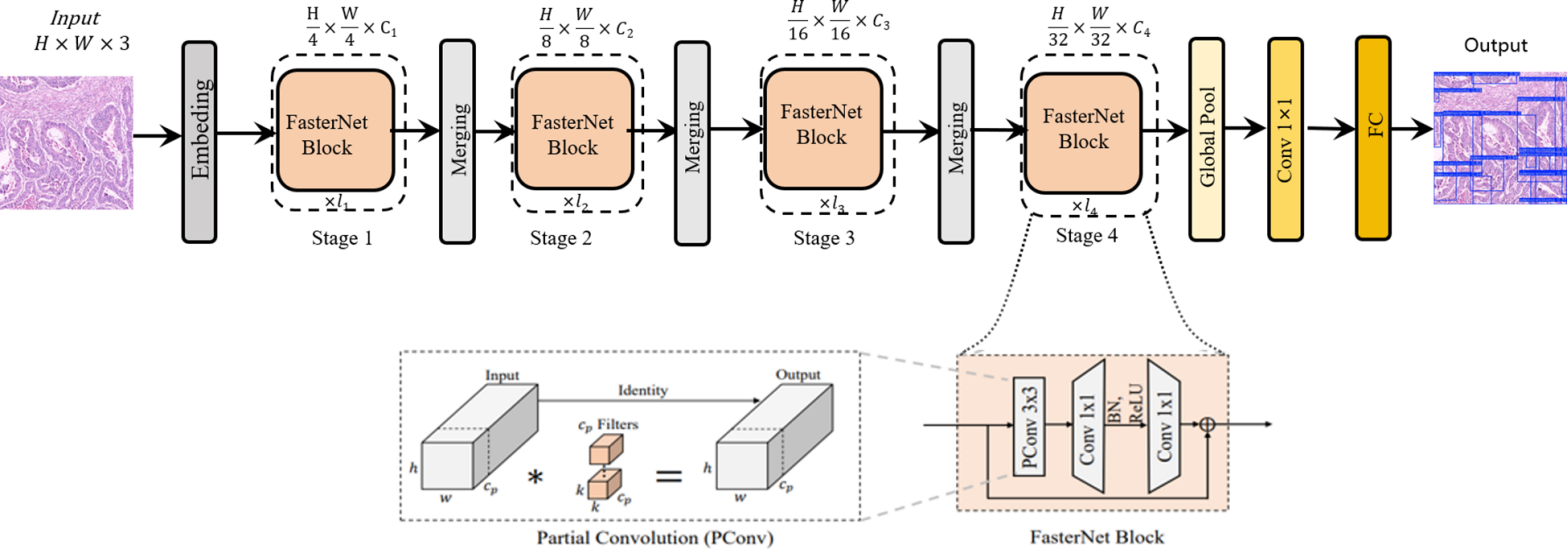
The overall architecture of FasterNet. The asterisk (*) in the “Partial Convolution (PConv)” diagram denotes the convolution.

While the standard backbone in YOLOv11s is effective, it represents a general-purpose design. When applied to pathological images—which are characterized by complex backgrounds and a high degree of similarity and redundancy between adjacent regions or across different feature map levels—this generic approach can lead to exhaustive convolutional operations. This results in an inefficient allocation of computational resources and a constrained capacity for extracting critical features. To address this limitation, we introduce the FasterNet network architecture. In contrast to standard convolutional backbones, FasterNet is architected around the principle of Partial Convolution (PConv), a design that reduces redundant computations, thereby enhancing computational efficiency while simultaneously promoting hardware-friendly implementation. This novel neural network family demonstrates exceptional computational efficiency and robust performance across diverse vision tasks. The architecture prioritizes hardware-friendly design through structural simplification, as illustrated in **Figure 1**. The framework comprises four hierarchical levels, each initiated with either an embedding layer (4×4 convolution with stride 4) or a merging layer (2×2 convolution with stride 2) for spatial downsampling and channel expansion. Each stage incorporates multiple FasterNet modules, with increased module density in the final two stages where memory access costs diminish and computational intensity (FLOPS) escalates. The core FasterNet module employs a partial convolution (PConv) layer followed by two point-wise convolutional (PWConv) layers, forming an inverted residual block with expanded intermediate channels and skip connections for feature reuse. Strategic architectural optimizations include: 1) Streamlined deployment of normalization and activation layers (post-PWConv only) to balance feature diversity with reduced inference latency; 2) Preferential use of batch normalization (BN) over layer normalization (LN) to leverage operator fusion capabilities with adjacent convolutional layers; 3) Dynamic resource allocation through multi-scale feature fusion - preserving moderate computational capacity in shallow layers for microscopic pattern extraction while intensifying FasterNet module density in deeper layers for macroscopic feature interpretation. These design principles collectively enhance detection accuracy while maintaining computational efficiency, particularly crucial for high-resolution pathological image analysis (17).

### 2.3 FC-YOLO for attention

**Figure 2:**
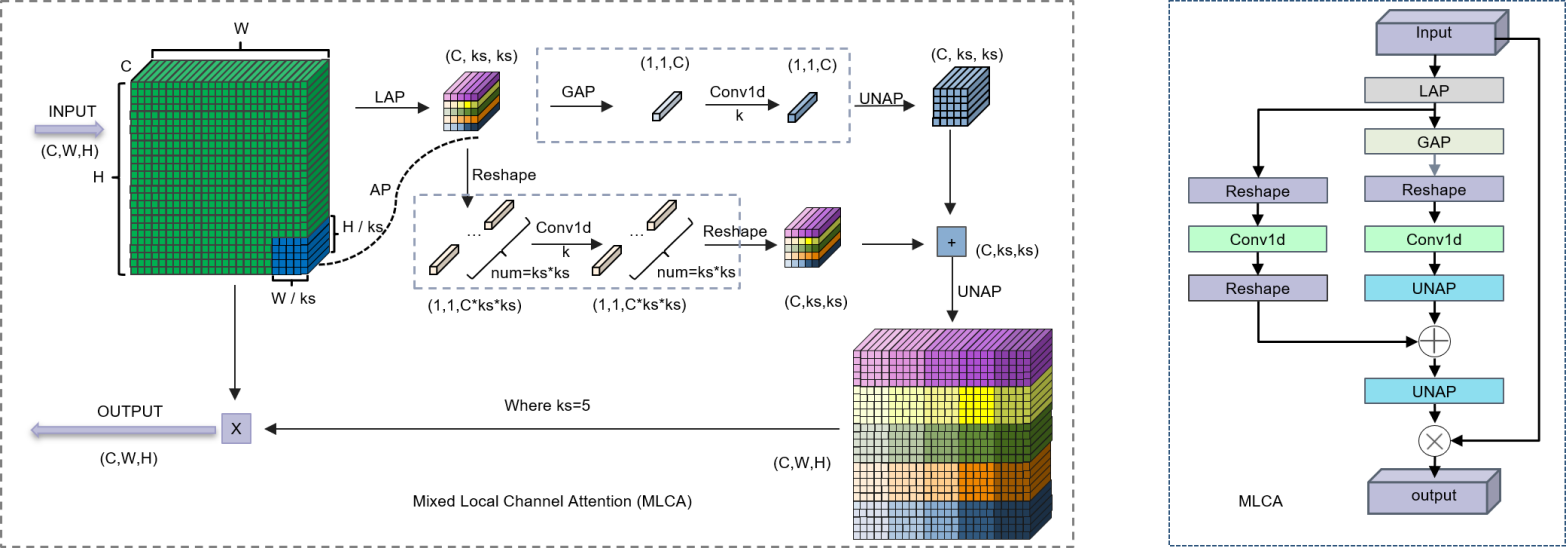
Left: Schematic diagram of the MLCA. Right: The structure of the MLCA. LAP (Local Average Pooling) which divides the feature map into k * k patches and applies average pooling to each patch; GAP (Global Average Pooling), which uses adaptive pooling to reduce the feature map to a 1 * 1 output size; UNAP (Anti-average Pooling), which mainly focuses on the figures) properties and scaling to the needed size.


The complex backgrounds and irregular target morphologies inherent to pathological images pose significant challenges to conventional attention mechanisms. Traditional attention methods, which establish channel-wise dependencies through global feature compression, often neglect critical local spatial context, thereby limiting their capacity to effectively represent features of heterogeneous lesions. While a recent study by Zubair et al. (18) proposed a multi-channel attention framework that enhanced the effectiveness and comprehensiveness of feature extraction in gastric cancer pathology images, the integration of this framework results in excessively high computational complexity. The parallel multi-channel computations and the stacking of attention mechanisms contribute to increased inference latency. Furthermore, this approach necessitates high-performance GPUs, rendering its deployment challenging in primary hospitals with limited resources. To address these limitations, we introduce a lightweight hybrid local attention mechanism, the Multi-scale Large-kernel Convolutional Attention (MLCA), which we integrate into the model’s backbone network. This innovative design enables efficient channel-spatial joint attention modeling with local-global feature synergy under low computational budgets. As depicted in **Figure 2**. The core principle of the MLCA mechanism is predicated upon a two-stage pooling process. Initially, the mechanism converts input features into a vector of dimensions 1 × C × ks × ks via a local pooling operation, designed for the efficient capture of local spatial information. Subsequently, feature extraction proceeds along two parallel branches: one branch is dedicated to extracting global contextual information, while the other focuses on preserving fine-grained local spatial details. The one-dimensional vectors generated by these branches are then processed by a 1D convolution (Conv1d), after which their resolution is restored to the original dimensions. A final information fusion step realizes the objective of the hybrid attention mechanism. Notably, the kernel size, *k*, of the 1D convolution is proportional to the number of channels, C. Its primary function is to capture local cross-channel interactions between each channel and its *k* adjacent neighbors. The formula for computing *k* is provided in Equation 1, where *k* represents the kernel size, C is the channel count, and γ and b are hyperparameters with default values of 2. To ensure that the kernel size *k* is always odd, it is incremented by one if the calculated value is even. Furthermore, the MLCA mechanism simultaneously considers channel information, spatial dimensions, and multi-level features from both local and global perspectives. This design effectively addresses a common limitation of traditional channel attention mechanisms—the neglect of spatial feature details—thereby reducing the computational burden of spatial attention modules while enhancing the model’s representational power and detection performance. Through the application of two-stage pooling and a dynamically optimized 1D convolution, MLCA not only improves processing speed but also circumvents the potential accuracy degradation associated with channel dimensionality reduction. Ultimately, MLCA strikes an ideal balance between model complexity and performance gain, significantly enhancing both the scalability of the attention mechanism and the efficacy of object detection (19). Consequently, the MLCA mechanism has the potential to improve the detection sensitivity of gastric adenocarcinoma foci by suppressing staining artifacts and interference from inflammatory cells, all while maintaining computational efficiency.

(1) $$k=\Phi (C)=❘\frac{{\text{log}}_{2}C)}{\gamma }+\frac{b}{\gamma }{❘}_{odd}$$

### 2.4 CARAFE for FC-YOLO

In histopathology, the precise delineation of lesion boundaries is of paramount importance for diagnosis. However, the feature fusion module in YOLOv11s utilizes conventional upsampling methods, which are characterized by fixed convolutional kernel weights that remain agnostic to the input content. This static approach can result in the imprecise demarcation of cancer cell boundaries within pathological images and may also lead to suboptimal contextual awareness. To address these limitations, we implement CARAFE, which enhances contextual perception through large receptive fields (up to 5×5) and dynamically predicts upsampling kernel weights based on feature semantics. This adaptive mechanism significantly improves the model’s capability to exploit discriminative features in histopathological images with complex backgrounds, particularly for detecting subtle malignant patterns obscured by stromal interference (20).

**Figure 3:**
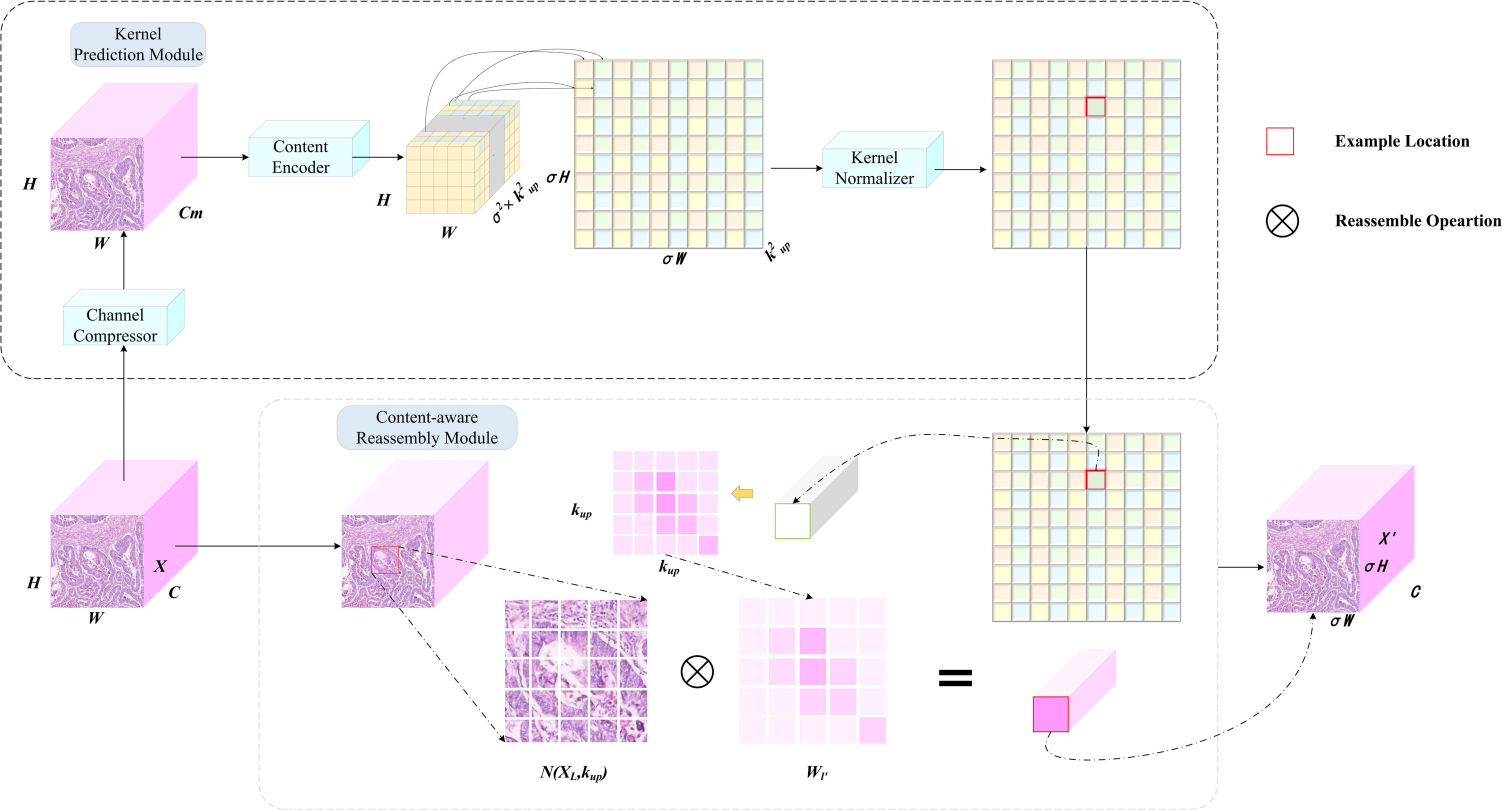
Work principles of CARAFE. CARAFE is composed of two key components, kernel prediction module and content-aware reassembly module.

The CARAFE module comprises two synergistic components: an upsampling kernel prediction module and a feature reassembly module, with its primary workflow illustrated in **Figure 3**. In the initial stage, the input feature map χ which contains target location information, undergoes channel compression. Subsequently, these compressed features are fed into a lightweight content encoder that dynamically predicts a unique reassembly kernel for each location. These kernels essentially define a set of weights for the neighboring pixels within the original feature map. A kernel normalization module then applies the softmax function to these weights, transforming them into a probability distribution. This mechanism enables the dynamic enhancement of minute details, such as alterations in cellular morphology, while simultaneously suppressing less relevant regions. In the second stage, based on a given upsampling rate σ, the reassembly kernels predicted in the preceding stage are applied to the original feature map. Through a weighted reassembly of information from the local neighborhood of the original feature map, a new feature map χ’ with dimensions of C × σH × σW is generated, thereby accomplishing the upsampling task. The integration of the CARAFE upsampling module enhances the feature extraction and fusion capabilities of the network’s neck, effectively mitigating detection challenges posed by complex background interference and densely distributed small targets, such as cancer cell clusters, within pathological images.

The CARAFE upsampling procedure operates through a systematic pipeline to achieve feature map resolution enhancement. Initially, the input feature map χ with dimensions H×W×C undergoes channel compression via a 1×1 convolutional layer, reducing the channel depth from C to C_m (where C_m < C) to optimize computational efficiency. Subsequently, a content encoder comprising convolutional layers generates a reorganization kernel tensor of shape H×W×σ²×k_up², where σ denotes the upsampling factor and k_up represents the receptive field size governing feature recombination. This tensor is spatially expanded to dimensions σH×σW×k_up², followed by kernel normalization to ensure the summation of convolutional weights equals unity. Within the content-aware reassembly module, each spatial position (i’, j’) in the output feature map χ’ corresponds to a k_up×k_up neighborhood region centered at (i/σ, j/σ) in the input feature map. The output activation is computed as the dot product between the predicted kernel weights and the unfolded input features. Notably, kernel sharing is implemented across channels at identical spatial locations, effectively balancing parameter efficiency and feature discriminability. Through this mechanism, the upsampled feature map χ’ with dimensions σH×σW×C is reconstructed while preserving structural coherence and semantic granularity.

### 2.5 FC-YOLO

The FC-YOLO model proposed in this study employs a hierarchical feature processing mechanism. Following standardized preprocessing, input images are first fed into a FasterNet-based backbone for multi-scale feature extraction. The extracted deep features are then passed through an SPPF module, which fuses contextual information via multi-scale pooling. Subsequently, the features proceed to a C2PSA module, which incorporates Position-Sensitive Attention (PSA) to enhance feature extraction and selective residual connections to optimize gradient propagation. Finally, high-level semantic features are outputted by the MLCA attention mechanism. Within this stage, MLCA utilizes a local-global dual-path pooling strategy combined with dynamic weight allocation to intensify the feature response to atypical cells.

**Figure 4:**
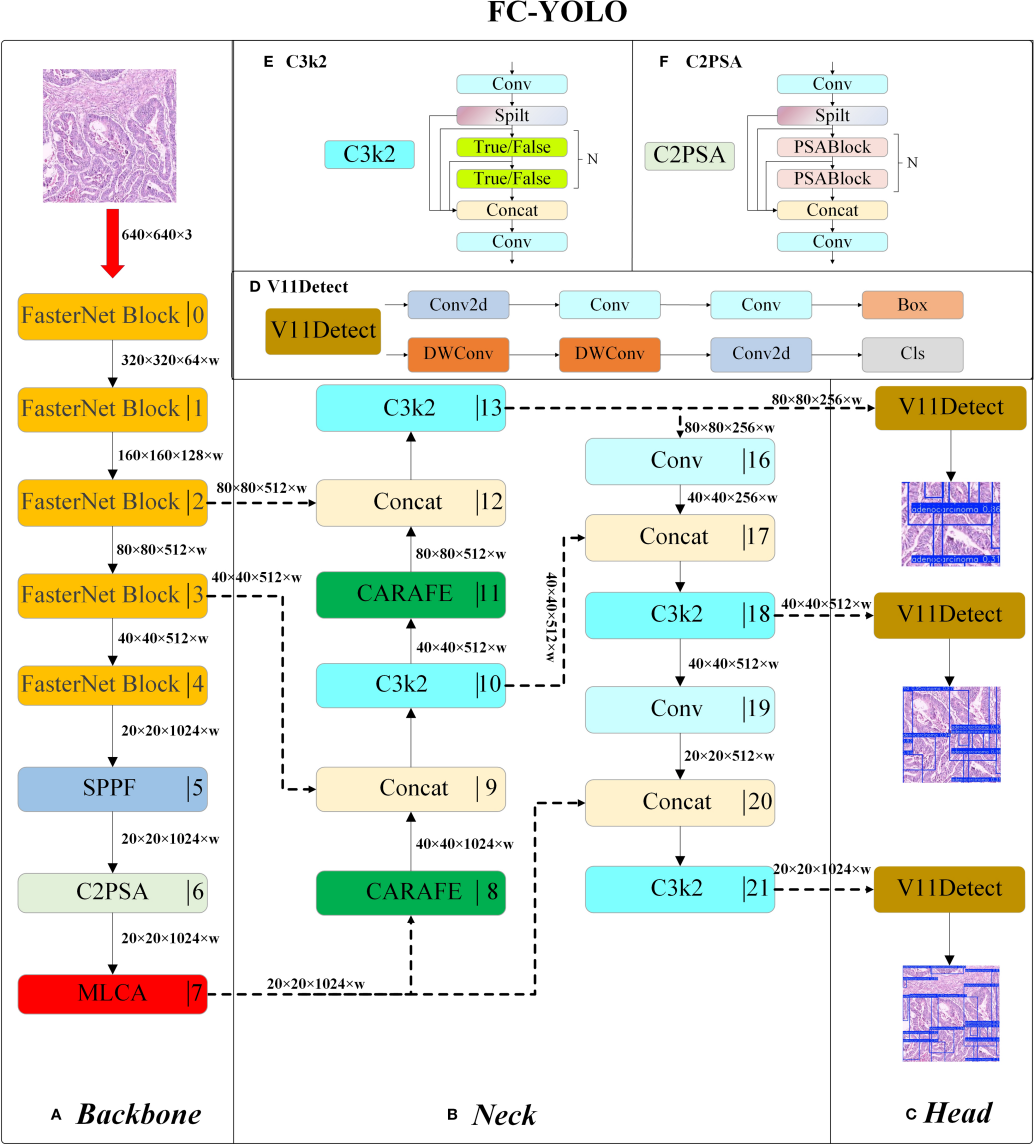
The structure of FC-YOLO: Backbone **(A)**, Neck **(B)**, Head **(C)**, V11Detect **(D)**, C3k2 **(E)**, and C2PSA **(F)**.

The enhanced neck branch adopts a bi-directional feature pyramid architecture to promote multi-scale information interaction. On the upsampling path, features are first upsampled by a CARAFE module and concatenated along the channel dimension with the output from Stage3 of the backbone. These fused features are then refined by a C3k2 module with cross-stage residual connections. This process is repeated: the refined features are again upsampled by CARAFE and fused with features from Stage2 of the backbone. Throughout this process, CARAFE’s dynamic kernel generation mechanism effectively recovers the geometric structural features of minute lesions by modeling local context. The downsampling path utilizes 3×3 standard convolutions with a stride of 2 to progressively compress spatial dimensions. Features at an 80×80 resolution are downsampled to 40×40, concatenated with the refined features from the first level of the upsampling path, and then fed into a C3k2 module to achieve cross-scale semantic fusion. A further downsampling to 20×20 resolution follows, where the features are concatenated with the original MLCA-enhanced high-level features, thereby constructing a feature pyramid rich in multi-dimensional semantic information. The C3k2 module optimizes the parameter count by employing bottleneck structures and depth-wise separable convolutions, reducing the computational load while preserving representational capacity. Inference is ultimately performed synergistically by multi-scale detection heads. The entire architecture achieves efficient detection of multi-scale lesions in gastric cancer pathology images through the synergistic interplay of MLCA’s cross-dimensional attention, CARAFE’s dynamic content-aware upsampling, and FasterNet’s high-efficiency feature extraction. A detailed illustration of the model architecture is provided in **Figure 4**.

### 2.6 The fabrication of the dataset

Two distinct datasets were utilized in this study: the BOT Dataset and In-House Clinical Dataset. The BOT Dataset, initially comprising 700 gastric adenocarcinoma images, was expanded to a total of 1,855 images through random data augmentation and preprocessing. This expanded collection, serving as our internal dataset, was subsequently partitioned into training, validation, and test sets at an 8:1:1 ratio, resulting in 1,485 images for training, 175 for validation, and 184 for testing. The In-House Clinical Dataset was constructed from 2,500 pathological images of gastric adenocarcinoma, acquired via microscopic examination from samples of 50 patients at the Affiliated Hospital of Baotou Medical College, Inner Mongolia University of Science and Technology, between 2018 and 2023. This dataset was also partitioned according to an 8:1:1 ratio, yielding 2,000 images for the training set and 250 images each for the validation and test sets. **Table 1**. provides a detailed summary of this dataset partitioning.

**Table 1:** The partitioning of the dataset.

Name	Dataset	Data source	Proportion	Number of pictures
BOT Dataset	training set	The 2017 China Big Data Artificial Intelligence Innovation and Entrepreneurship Competition	80%	1485
	validation set		10%	175
	test set		10%	184
In-House Clinical Dataset	training set	Affiliated Hospital of Baotou Medical College, Inner Mongolia University of Science and Technology	80%	2000
	validation set		10%	250
	test set		10%	250

### 2.7 Evaluation indicators

The trained model was applied to the test set, generating predictions that included bounding box coordinates and confidence scores. Confidence scores, ranging from 0 to 1, quantify the probability of a predicted bounding box belonging to a specific category, with higher values indicating greater certainty.

To rigorously evaluate prediction accuracy, seven metrics were employed:

1. Precision (P): Ratioof true positives (TP) to all positive predictions and the calculation method is shown in Equation 2.


(2) $$\text{Precision}=\frac{TP}{TP+FP}$$

2. Recall (R):Ratio of true positives to all actual positives and is calculated using Equation 3.


(3) $$\text{Recall}=\frac{\text{TP}}{\text{TP}+\text{FN}}$$

3. mAP@50: Mean average precision at an intersection-over-union (IoU) threshold of 0.5, the calculation formula is shown in Equation 4.


(4) $$\text{mAP}=\frac{\sum_{1}^{\text{m}}\int_{0}^{1}\text{p}(\mathbb{R})\text{dR}}{\text{N}}$$

4. mAP@50-95: Mean average precision averaged over IoU thresholds from 0.5 to 0.95 (step: 0.05).

5. Frames Per Second (FPS): Real-time inference speed.

6. F1 Score: Harmonic mean of precision and recall, computed as indicated in Equation 5.


(5) $$\text{F}1=2\times \frac{\text{Precision}\times \text{Recall}}{\text{Precision}+\text{Recall}}$$

7. Operations Per Second (GFLOPS): Computational complexity in giga floating-point operations per second.

### 2.8 Model parameter configuration and training

The hyperparameter settings for the training are shown in **Table 2**.

**Table 2:** Parameter settings.

Epoch	Batch_size	Initial-learning rate	Optimizer	Lr decay
600	32	0.01	Adam	Step

Hyperparameter configurations were executed through a command-line interface (CLI). Upon initiating the training command, the model training process commenced. The program first loaded the predefined model architecture, followed by iterative training on the dataset. After completing 600 epochs, the training phase concluded, and the finalized model was saved to a user-specified directory path. Throughout this workflow, identical hyperparameter settings and training protocols were applied to all YOLOv11 model variants (e.g., YOLOv11n, YOLOv11s), ensuring consistency in comparative performance evaluation.

### 2.9 Data expansion

The histopathological images in both datasets for this study were annotated using the Labelme software. This was a two-tiered process: the initial annotations were performed by two physicians with over five years of experience in pathological diagnosis. To ensure accuracy, any annotations considered ambiguous were subsequently reviewed and finalized by a senior pathologist with more than ten years of diagnostic experience. This structured workflow was designed to minimize annotation bias and ensure the high quality of the ground-truth labels. To address the significant stain variations in histopathological images, we considered both standard normalization techniques and a more advanced component-wise approach. Standard methods, such as those proposed by Reinhard et al. (21). and Macenko et al. (22)., are effective for global stain normalization by mapping the color space of an image to a target template. However, for gastric adenocarcinoma diagnosis, where subtle nuclear atypia and chromatin texture (stained by hematoxylin) are critical diagnostic features, a global transformation risks obscuring these minute but vital local details. Moreover, such a global stain normalization approach is detrimental to enhancing the model’s generalization capability and its robustness against the inherent variations present in pathological images. Consequently, we adopted the more sophisticated method proposed by Tellez et al. (23). This approach was chosen to ensure that the augmented images could more accurately reflect both the cellular atypia and the image transformations arising from staining variations inherent in clinical practice, thereby enhancing the model’s detection performance. Specifically, our data augmentation pipeline began with a series of spatial transformations, including random rotations, horizontal and vertical flips, isotropic scaling sampled from a uniform distribution, and elastic deformations (with parameters α=100, σ=10) designed to simulate minor tissue distortions. Subsequently, the RGB image patches were converted into the Hematoxylin (H), Eosin (E), and Residual (R) color space. The intensity of each channel was then perturbed using an independent multiplicative factor α, sampled from a uniform distribution [0.95, 1.05]. and an additive bias β, sampled from [−0.05, 0.05]. After reconstructing the images back into the RGB space, we introduced further variations by randomly adjusting their brightness and contrast with factors sampled from a uniform distribution of [0.75, 1.5]. and their color saturation with factors from [0.75, 1.25]. Finally, a Gaussian blur of random intensity was applied to a subset of these images. The specific parameters and their corresponding distributions are detailed in **Table 1** of Appendix 1.

### 2.10 Visualization of the training network

The GradCAM++ method (24) was utilized to analyze the trained network, providing visual insights into the basis of the predictions.

### 2.11 Software and hardware

The computational framework was implemented using PyTorch 1.13.1. All experimental procedures - including data pipeline construction, preprocessing operations, network training, and inference tasks - were executed on a high-performance workstation equipped with an Intel Core i7-12700K CPU (3.20GHz base frequency, 4.40GHz turbo boost), 128GB DDR4 RAM, and dual NVIDIA GeForce RTX 4090 D GPUs with 24GB GDDR6X memory each.

## 3 Experiments and results

### 3.1 Comparison of basic models

The selection of an appropriate baseline model is a critical step in the development of computer-aided diagnosis systems intended for practical clinical application. Our choice of YOLOv11s as the base architecture was informed by a multi-faceted evaluation of its detection accuracy on pathological images, inference speed, and computational efficiency, the latter being a proxy for deployment feasibility. While numerous YOLO variants exist, such as the widely adopted YOLOv5 and the powerful YOLOv8, we specifically selected YOLOv11 (25).as it represents the latest advancements in optimizing the accuracy-efficiency trade-off for real-time applications. To substantiate this choice, we conducted a preliminary comparative analysis of the YOLOv11 variants (**Table 3**). This comparison illuminated a fundamental principle: larger models (e.g., YOLOv11m/l/x) deliver superior accuracy but at a substantial computational cost, rendering their deployment impractical in many clinical settings with limited hardware resources. Conversely, the smallest model (YOLOv11n), while highly efficient, lacks the requisite accuracy for high-stakes diagnostic tasks, where a missed detection could have severe consequences. YOLOv11s, however, strikes a judicious balance. It offers a significant improvement in accuracy over YOLOv11n while maintaining a modest parameter count (9.45M) and a low computational load (21.7 GFLOPS). This “sweet spot” is paramount; it establishes a robust performance baseline that is sufficiently high for meaningful clinical assistance, yet efficient enough to permit real-time inference on standard GPUs. This equilibrium makes it an ideal foundation for our subsequent optimizations. Furthermore, compared to other object detection models, YOLOv11s incorporates state-of-the-art architectural improvements, providing an advantageous starting point for the integration of our custom modules: FasterNet, MLCA, and CARAFE. Consequently, YOLOv11s was chosen as the foundational model for our study.

**Table 3:** Comparison of different versions of YOLOv11.

Models	mAP50(%)	Params[m]	GFLOPS
YOLOv11n	78.2	2.62	6.6
YOLOv11s	80.3	9.45	21.7
YOLOv11m	82.1	20.1	68.5
YOLOv11l	83.9	25.3	87.6
YOLOv11x	84.2	56.9	196

### 3.2 The comparison of accuracy among different backbone networks

To identify optimal CNN backbone architectures, we conducted a systematic evaluation of classical deep learning models (VGG16 (26). ResNet50 (27). MobileNetV2 (28). FasterNet, and EfficientViT (29)) under standardized experimental protocols. **Table 4** presents their validation performance in gastric adenocarcinoma detection tasks with data augmentation. The reported metrics represent mean accuracy scores derived from four-fold cross-validation. A comparative analysis revealed that FasterNet, ResNet50, and EfficientViT demonstrate comparable performance. Consequently, these architectures were selected to replace the backbone component of the YOLOv11 model. A subsequent comparative study was conducted to further investigate which of these CNN architectures is most adept for object detection tasks within the context of histopathological analysis.

**Table 4:** The comparison among backbone networks.

CNN architecture	Validation accuracy
VGG16	0.757
ResNet50	0.763
FasterNet	0.766
EfficientNetViT	0.768
MobileNetV2	0.755

**Figure 5:**
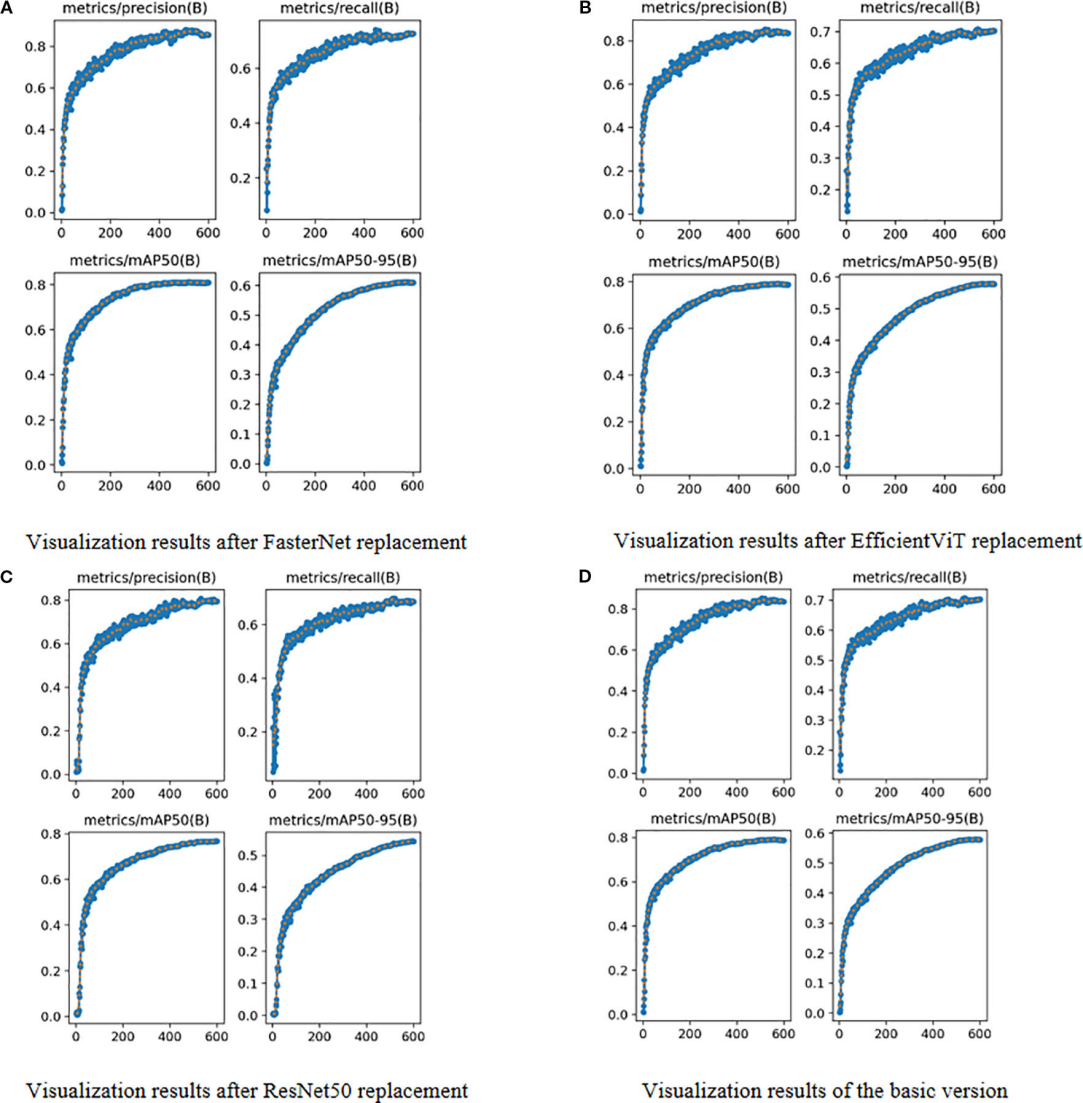
Visual Comparison of Different Backbone Networks. **(A)** Visualization results after FasterNet replacement. **(B)** Visualization results after EfficientViT replacement. **(C)** Visualization results after ResNet50 replacement. **(D)** Visualization results of the basic version.

During the training phase, it is crucial to monitor the evolution of precision and recall, while the final training outcomes are assessed using mAP@0.5 and mAP@[0.5:0.95]. These metrics offer valuable insights into a model’s performance and its generalization capability. As detailed in **Table 5** and **Figure 5**, we evaluated three high-performing backbone networks—FasterNet, EfficientViT, and ResNet50—by substituting them into the YOLOv11s architecture. The analysis of the results indicates that, compared to the baseline version, the FasterNet substitution yielded the most favorable performance. Specifically, it achieved a 1.2% increase in mAP and a 1.9% improvement in accuracy. Consequently, based on these findings, FasterNet was definitively selected as the backbone for our model.

**Table 5:** Gastric cancer indicators under different models.

Models	P	R	mAP50	mAP50-95
Baseline(YOLOv11s)	0.835	0.713	0.802	0.577
FasterNet	0.854	0.715	0.814	0.615
Efficientvit	0.841	0.710	0.806	0.578
ResNet50	0.795	0.682	0.794	0.544

### 3.3 Attention mechanism compatibility experiment

To validate the architectural compatibility of MLCA, we conducted comparative experiments with five prevalent attention mechanisms (GAM (30). BiFormer (31). CA (32). CPCA (33). SimAM (34). CBAM (35)) integrated into the baseline model. The Grad-CAM++ visualization technique was systematically applied to generate class-discriminative localization maps, enabling quantitative assessment of spatial attention distribution variations across different attention-enhanced architectures. This analytical approach elucidates region-specific feature prioritization patterns and establishes interpretable correlations between attention-driven feature selection and diagnostic performance metrics.

**Figure 6:**
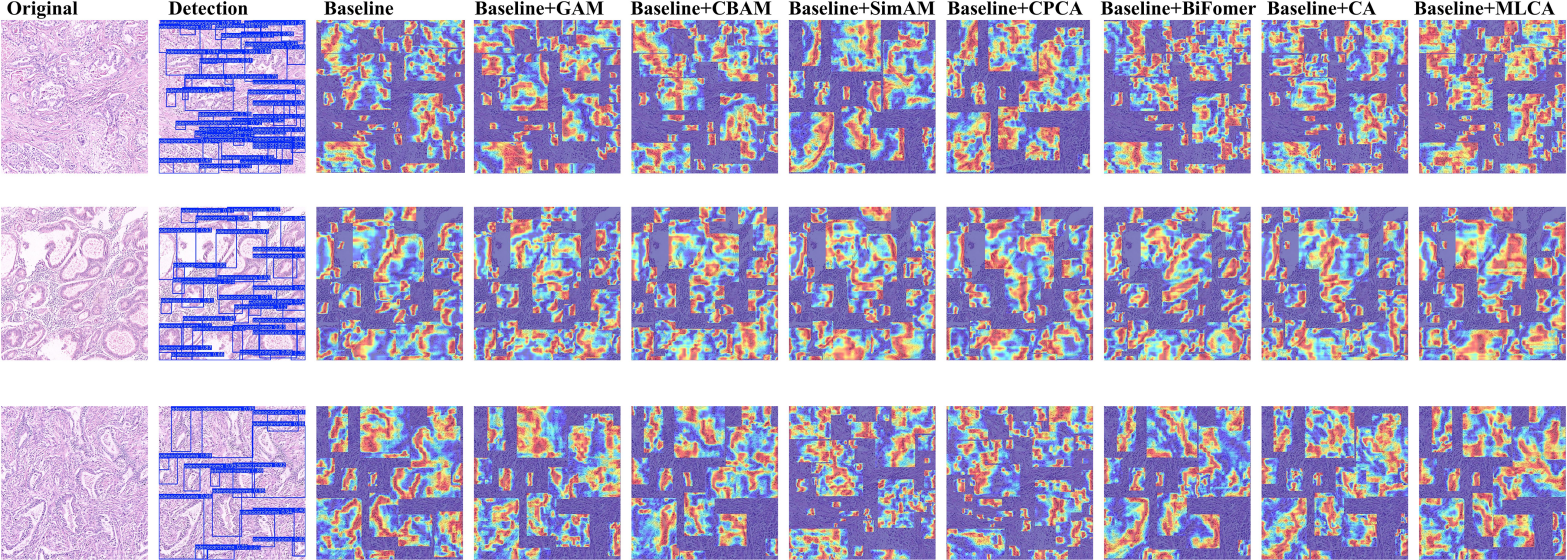
Heatmaps for various attention mechanisms.

As shown in **Figure 6**, our comparative experiments revealed that the MLCA attention mechanism outperforms conventional modules like CBAM and CA. Specifically, MLCA generates heatmaps with significantly expanded spatial regions of interest, indicating its ability to guide the model in capturing multi-scale features from target regions. By fusing local details with global contextual information, MLCA creates a comprehensive representation of the Region of Interest (ROI), a capability crucial for detecting minute lesions in pathological images. Furthermore, the high-activation regions (indicated in red) generated by MLCA are more extensive, suggesting an enhanced capacity for extracting salient features. We attribute this advantage to MLCA’s unique dual-path weight allocation strategy. One path captures multi-resolution features via an atrous convolution pyramid, while the other employs channel-spatial joint attention to filter critical information. This dual approach effectively suppresses interference from complex tissue backgrounds, such as stromal fibrosis and inflammatory infiltration. Consequently, MLCA demonstrated superior performance in detecting gastric adenocarcinoma in pathological images compared to the other attention mechanisms tested.

Subsequently, we conducted a quantitative analysis using the mean Average Precision (mAP) evaluation metric. In this ablation study, only the attention mechanism module was systematically varied, while other components remained constant, followed by the measurement of the mAP value for each resulting model configuration. This methodology allowed for a direct comparison of mAP scores across different models, thereby enabling an assessment of the compatibility and efficacy of various attention mechanisms within the base architecture. The comparative experimental data are presented in **Table 6**. The results demonstrate that the model variant equipped with the MLCA attention mechanism achieved superior detection performance (mAP) compared to both the baseline version (YOLOv11s without an additional attention module) and variants incorporating alternative attention mechanisms, specifically GAM, CBAM, SimAM, CPCA, BiFormer, and CA. Notably, we observed that the integration of the GAM and SimAM attention mechanisms resulted in a decrease in mAP by 0.3% and 0.7%, respectively, relative to the baseline. Conversely, incorporating the CBAM, CPCA, BiFormer, and CA attention mechanisms yielded improvements in mAP by 0.1%, 0.3%, 0.5%, and 0.7%, respectively. Significantly, the fusion of the MLCA attention mechanism led to the most substantial performance gain, enhancing the mAP by 1.1%. These findings strongly suggest that our proposed model, featuring the MLCA attention mechanism, is better suited and more effective for the task of detecting gastric adenocarcinoma in pathological images compared to the original YOLOv11s baseline model.

**Table 6:** The comparison between different attention modules.

Model	Attention	F1(%)	mAP(%)
YOLOv11s	——	75.9	80.2
YOLOv11s	GAM	74.5	79.9
YOLOv11s	CBAM	75.2	80.3
YOLOv11s	SimAM	74.1	79.5
YOLOv11s	CPCA	74.2	80.5
YOLOv11s	BiFomer	75.8	80.7
YOLOv11s	CA	75.7	80.9
YOLOv11s	MLCA	77.1	81.3

### 3.4 Ablation experiment

We conducted ablation experiments to validate the impact of our three proposed improvements. As detailed in **Table 7**, we evaluated eight experimental configurations by comparing them against the baseline YOLOv11s model. The performance of each configuration was assessed using mAP, F1-score, Recall, and Precision. We designated the modified configurations based on the added components—for example, “YOLOv11s+FasterNet” for the FasterNet backbone, “YOLOv11s+CARAFE” for the CARAFE upsampling module, and “YOLOv11s+MLCA” for the MLCA attention mechanism. This naming pattern was applied consistently across all combinations.

**Table 7:** The impact of the fusion of different modules of the model on the metrics.

Model	mAP (%)	F1 (%)	Recall	Precision	FPS
Baseline					
YOLOv11s	80.2	76.9	0.713	0.835	125.78
Single Component Improvements					
YOLOv11s+FasterNet	81.4	78.2	0.705	0.854	129.23
YOLOv11s+CARAFE	80.4	76.2	0.71	0.821	130.12
YOLOv11s+ MLCA	81.3	77.2	0.712	0.843	124.42
Paired Component Improvements					
YOLOv11s+ MLCA+CARAFE	79.8	76.3	0.722	0.809	128.47
YOLOv11s+FasterNet+ MLCA	81.9	77.9	0.727	0.84	126.89
YOLOv11s+FasterNet+CARAFE	81.1	77.6	0.716	0.848	133.52
Final Model					
YOLOv11s+FasterNet+CARAFE+MLCA	82.8	79.8	0.745	0.861	131.56

As shown in **Table 7**, integrating the FasterNet backbone and the MLCA attention module into YOLOv11s improved accuracy by 1.2% and 1.1%, respectively. This result highlights FasterNet’s superior ability to extract multi-scale histopathological features, guiding feature attention toward diagnostically critical patterns and improving feature representation. Notably, the MLCA module illustrates a clear accuracy-speed tradeoff: it increased mAP by 1.1% and the F1-score by 0.3%, with only a marginal reduction in inference speed. Furthermore, integrating CARAFE significantly improved computational efficiency. Its adaptive upsampling mechanism resulted in a 4.6% increase in inference speed compared to the baseline model.

### 3.5 Comparison experiments with other mainstream object detection algorithms

**Figure 7:**
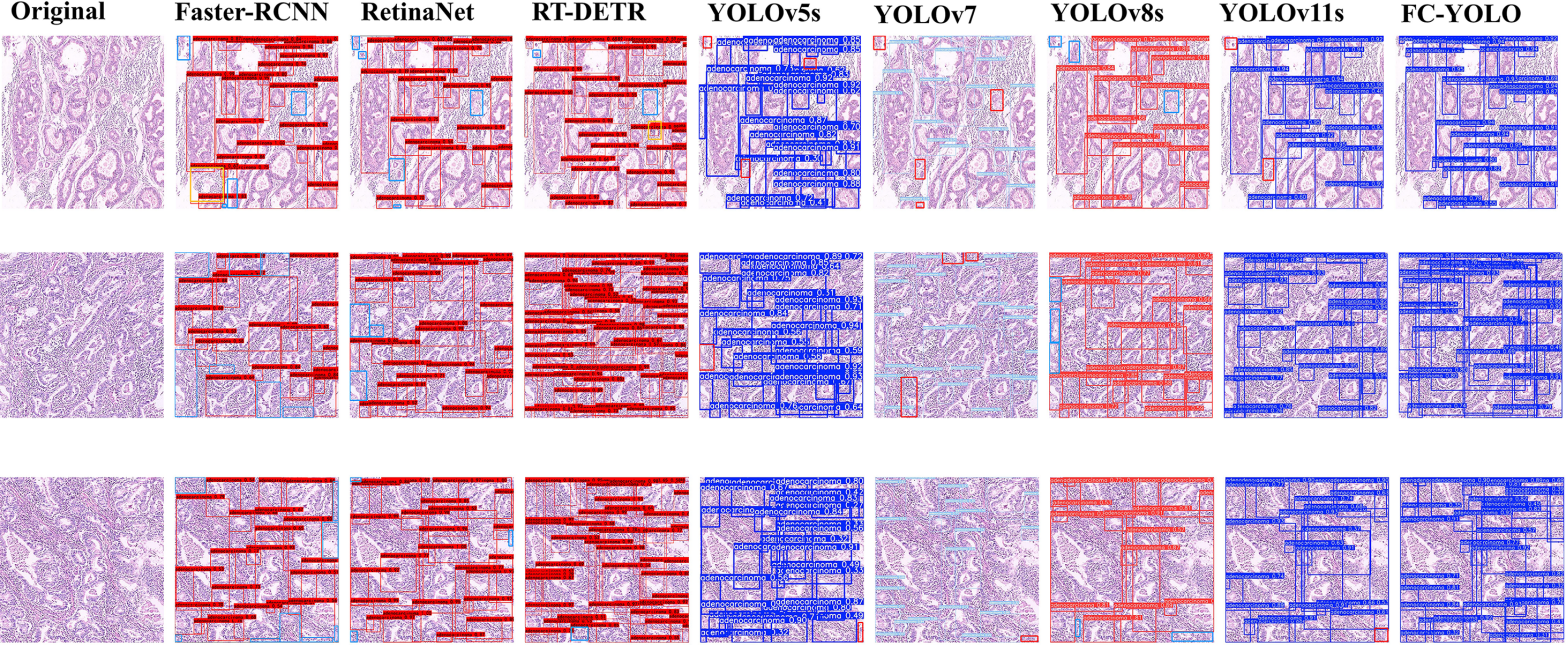
Compared with other mainstream object detection algorithms.

The proposed FC-YOLO algorithm enhances feature extraction from complex histopathological images, making it a high-performance solution for detecting gastric adenocarcinoma. To validate its performance, we compared it with several mainstream object detection architectures: Faster-RCNN (36). RT-DETR (37). RetinaNet (38). YOLOv5s (39). YOLOv7 (40). YOLOv8s (41). and the baseline YOLOv11s. To ensure a fair and valid assessment, we retrained each model from scratch under the same experimental conditions used for FC-YOLO. As detailed in **Table 8** and **Figure 7**, the comprehensive benchmarking results confirm the diagnostic efficacy of FC-YOLO, a finding supported by this rigorous methodological consistency.

**Table 8:** Different model variants.

Model	mAP (%)	FPS	F1 (%)	Params[m]	GLOPs
Faster-RCNN RetinaNet RT-DETR Yolov5s Yolov7 Yolov8s Yolov11s FC-YOLO	67.4 71.8 78.7 79.1 78.5 78.9 80.2 82.8	85.42 97.21 119.97 120.42 117.35 122.26 125.78 131.56	64.2 68.7 74.7 75.5 74.9 74.3 77.1 79.8	127.35 35.56 32.7 7.02 37.1 11.1 9.45 7.68	416.5 120.4 112.4 16.5 105.1 28.8 21.7 15.9

A qualitative analysis of the visual detection results (**Figure 7**) reveals distinct performance characteristics among the evaluated architectures. The transformer-based RT-DETR shows better detection consistency than Faster R-CNN and RetinaNet, with precision comparable to that of YOLOv5s. However, RT-DETR produces notable misclassification artifacts in histologically ambiguous regions. Faster R-CNN exhibits significant limitations, including both missed detections and classification errors. While YOLOv11s improves upon YOLOv5s by reducing missed detections, it still under-detects subtle pathological manifestations. In contrast, our proposed FC-YOLO architecture demonstrates an enhanced ability to discriminate microscopic lesions, particularly in challenging scenarios that require differentiating malignant glands (e.g., irregular lumen structures) from inflammatory infiltrates. These comparative evaluations confirm the diagnostic superiority of FC-YOLO, which reduces misclassifications while increasing true-positive detections relative to its state-of-the-art counterparts.

In our subsequent experimental evaluation, we used mean Average Precision (mAP), F1 score, Frames Per Second (FPS), parameter count, and Giga Floating-point Operations Per Second (GFLOPS) as key performance metrics. As detailed in **Table 8**, our proposed FC-YOLO algorithm achieved a state-of-the-art mAP of 82.8%. Compared to the YOLOv11s baseline, FC-YOLO improved the mAP by 2.6% and the F1 score by 2.7% (to 79.8%), indicating a superior balance between precision and recall. Crucially, these accuracy gains were achieved alongside significant efficiency improvements. The integration of the FasterNet backbone and CARAFE upsampling module allowed us to reduce the parameter count by 18.7% (from 9.45M to 7.68M) and the computational load by 26.7% (from 21.7 to 15.9 GFLOPS), while increasing inference speed by 4.6%.

Beyond the baseline, we benchmarked FC-YOLO against other mainstream architectures. It significantly outperformed classic algorithms, achieving a 15.4% higher mAP than Faster R-CNN and an 11% higher mAP than RetinaNet. It also surpassed the modern hybrid Transformer-based model, RT-DETR, with a 4.1% mAP enhancement. When comparing other YOLO versions, we noted an intriguing result: in the specific context of gastric adenocarcinoma detection, YOLOv5s unexpectedly outperformed both YOLOv7 and YOLOv8s by 0.6% and 0.2% mAP, respectively. This observation is corroborated by **Figure 7**, which shows YOLOv5s produced fewer missed detections. Nevertheless, our FC-YOLO model still achieved a 3.7% higher mAP than YOLOv5s, despite having a comparable parameter count.

**Figure 8:**
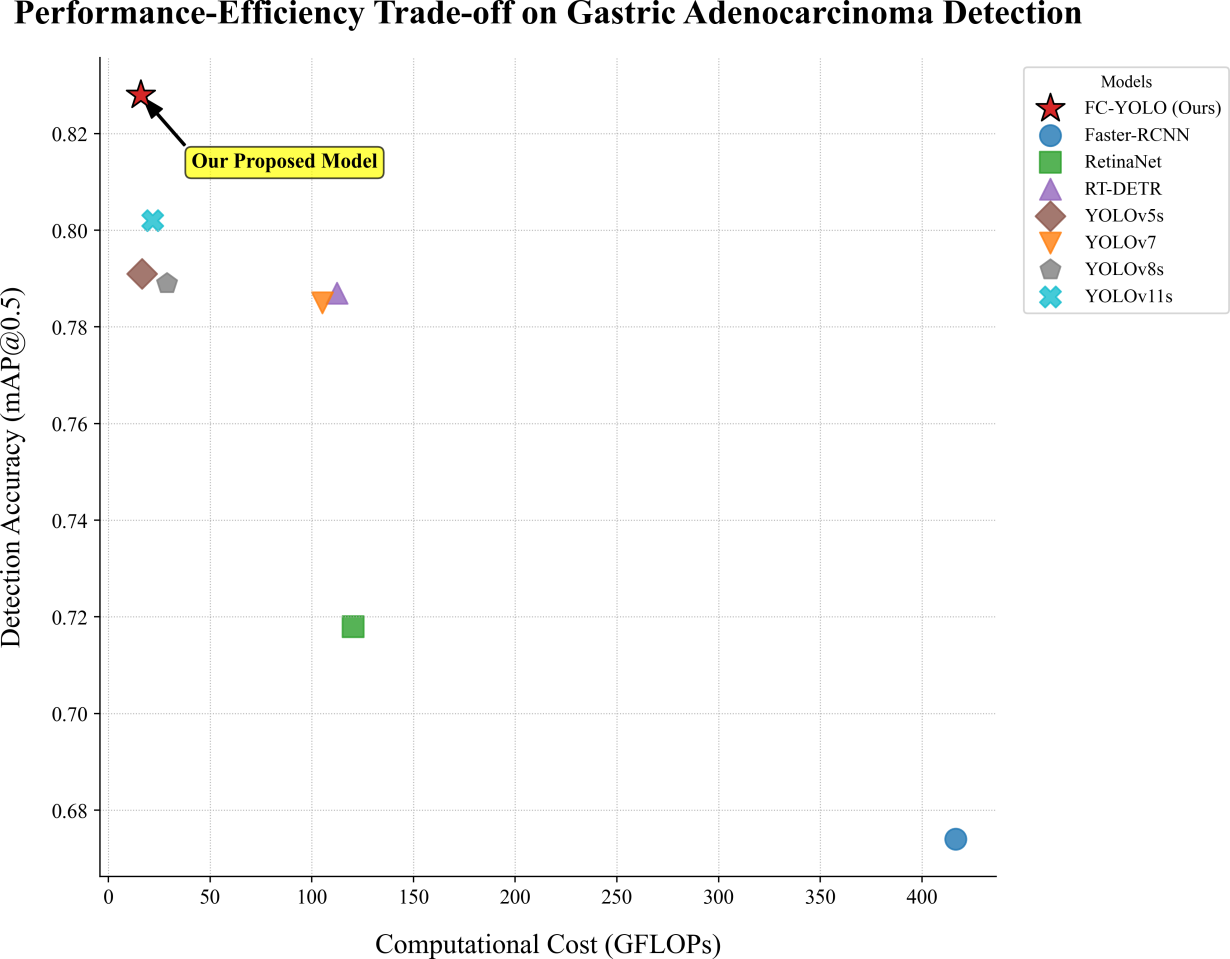
Accuracy-efficiency trade-offs.

These results underscore that FC-YOLO establishes a new state-of-the-art balance between diagnostic accuracy and computational cost, a critical factor for practical clinical deployment (**Figure 8**). This favorable trade-off is a direct result of our architectural choices. By replacing the standard backbone and upsampling modules with the more efficient FasterNet and CARAFE, we enhanced accuracy without increasing computational cost. The lightweight MLCA attention module further augmented the model’s discriminative power at a negligible cost. In conclusion, FC-YOLO demonstrates a significant advantage for detecting gastric adenocarcinoma in pathological images, achieving superior accuracy while maintaining a lower computational cost and faster inference speed than current mainstream algorithms.

**Figure 9:**
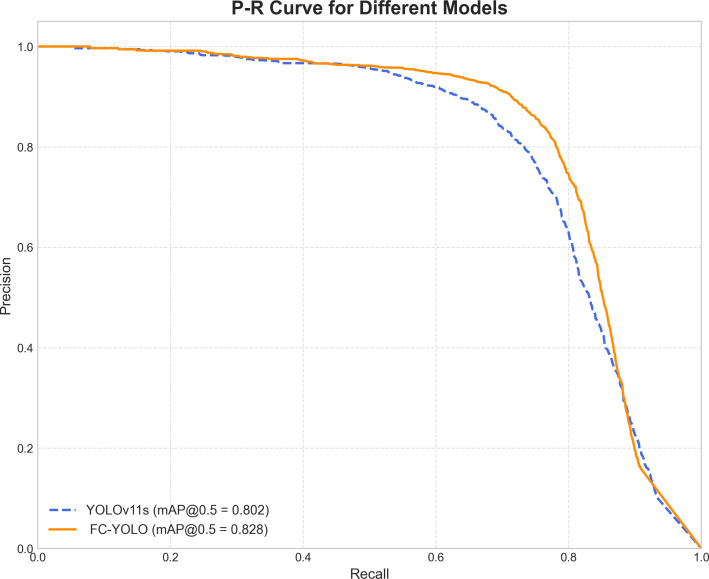
Comparison of the Precision-Recall (PR) curves for YOLOv11s and FC-YOLO.

**Figure 10:**
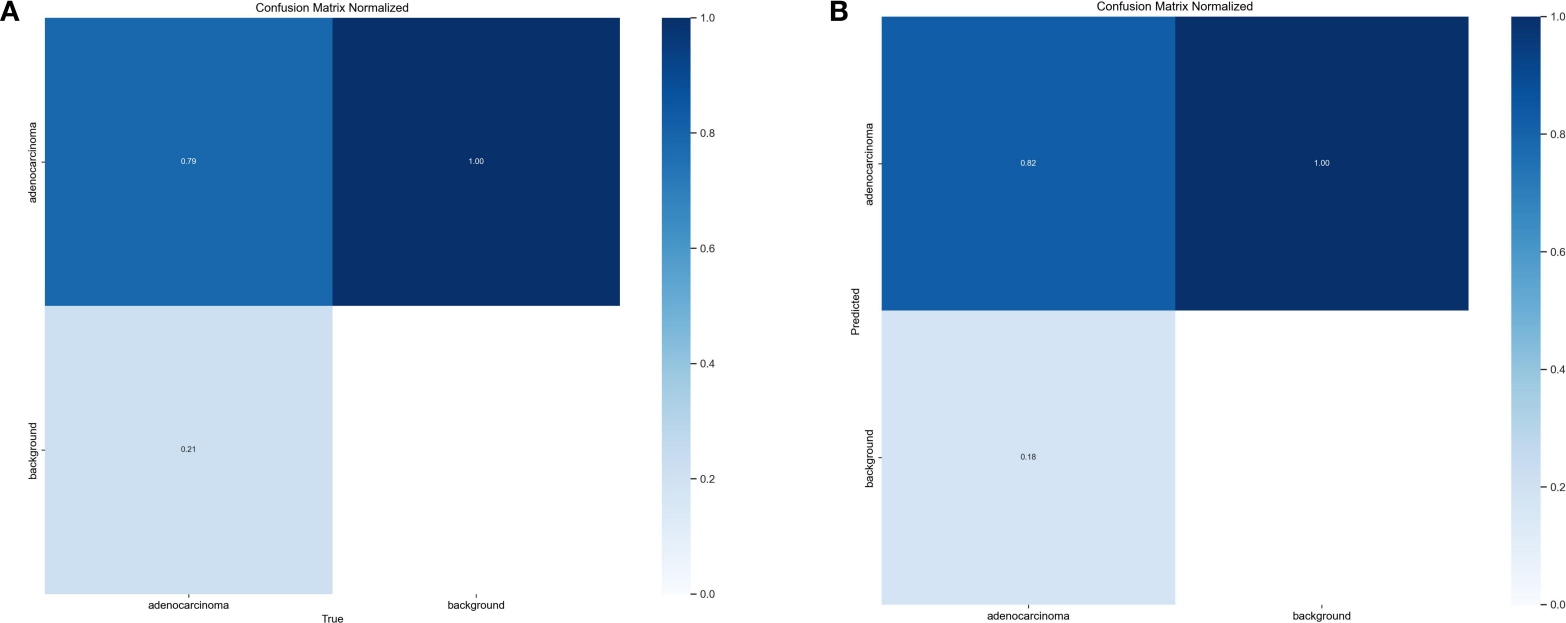
Confusion matrices. **(A)** Confusion matrix for the YOLOv11s model. **(B)** Confusion matrix for the FC-YOLO model.

To gain a deeper understanding of FC-YOLO’s diagnostic performance, we conducted a more granular analysis using Precision-Recall (PR) curves. These curves visualize the trade-off between precision and recall across various confidence thresholds, with a curve positioned closer to the top-right corner indicating superior performance. As shown in **Figure 9**, we compared the PR curves for FC-YOLO and the baseline YOLOv11s on the BOT dataset. The FC-YOLO curve is consistently positioned above the baseline, visually demonstrating that our model achieves higher detection sensitivity while being less prone to making erroneous predictions. We also used confusion matrices to evaluate classification performance by comparing true and predicted labels. In these matrices, superior performance is indicated by higher values along the diagonal and lower values in the off-diagonal cells. **Figures 10A,B** show the normalized confusion matrices for the baseline YOLOv11s and our FC-YOLO model, respectively, on the BOT dataset. A direct comparison reveals that FC-YOLO achieves higher detection accuracy, identifying gastric adenocarcinoma regions more precisely and with fewer misclassifications. In contrast, the baseline YOLOv11s model shows a higher incidence of both false positives and false negatives.

### 3.6 The verification of accuracy for In-House Clinical dataset

**Figure 11:**
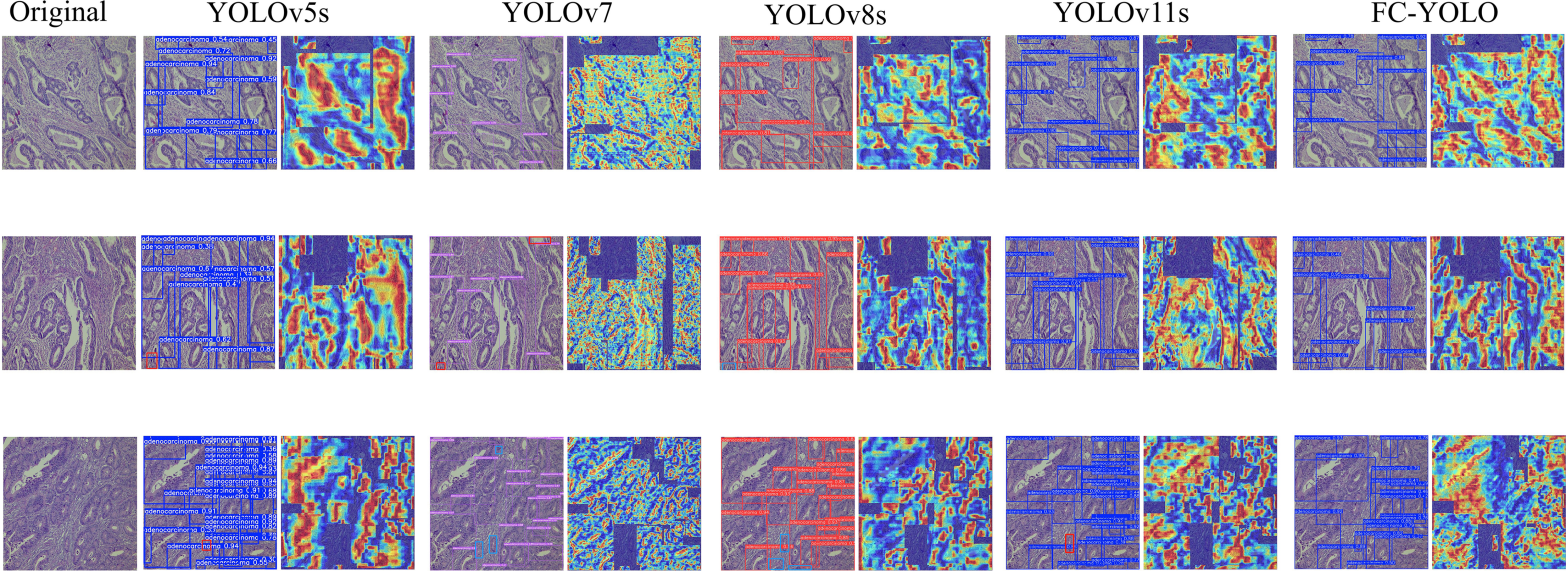
Prediction comparison chart.

Subsequently, the gastric adenocarcinoma pathological images collected from our hospital were utilized as an In-House Clinical Dataset, on which the model was trained from scratch. The objective was to ascertain whether our model’s enhancements retain their superiority for detecting gastric adenocarcinoma within a real-world clinical environment. A core challenge presented by such independent clinical dataset is the inherent variability in staining protocols and slide preparation. Acknowledging that performing independent stain normalization is often impractical and difficult to maintain consistently in a live clinical workflow, we deliberately omitted this step. Instead, the focus of our evaluation was shifted to assessing the model’s intrinsic robustness against these diverse staining schemes. The trained model was utilized to predict the collected gastric adenocarcinoma slices, and Grad-CAM heatmaps were conducted to verify the regions of interest of our model. Moreover, comparisons were made with yolov5s, yolov7, yolov8s, and yolov11s, which exhibited relatively superior performance in the control experiments, as depicted in **Figure 11**. It can be observed from the detection maps of different models that YOLOv7 and YOLOv8s present more missed detections during the detection process, especially in regions with complex backgrounds. Some missed detections are also found in YOLOv5s and YOLOv11s, particularly in the detection of small targets. In contrast, our model demonstrates a superior ability in target recognition of gastric adenocarcinoma. From the heatmaps, it can be noticed that the regions that our model focuses on are mostly the cancer cavities and areas with significant cellular atypia, which are more consistent with the characteristics of gastric adenocarcinoma. Furthermore, we also tested the inference speed of FC-YOLO in the clinically collected slices. As presented in **Table 9**. Different model variants., the inference speed and accuracy of our model surpass those of other YOLO series models.

**Table 9:** Different model variants.

Model	mAP	Precision	FPS	F1(%)
YOLOv5s	81.3	0.864	105.73	80.7
YOLOv7	80.4	0.847	102.62	78.3
YOLOv8s	80.1	0.856	108.98	79.5
YOLOv11s	82.9	0.878	113.58	81.5
FC-YOLO	85.7	0.897	121.77	82.3

### 3.7 Cross-dataset validation experiment

To rigorously evaluate the generalizability and robustness of our proposed FC-YOLO model, we conducted a cross-dataset validation experiment. This experiment utilized our two independent datasets: the BOT dataset and an In-House Clinical dataset. Both FC-YOLO and the baseline YOLOv11s adhered to a strictly consistent cross-validation protocol. In the first arm, models were trained exclusively on the BOT dataset and then directly evaluated on the validation set of the In-House Clinical dataset. Conversely, in the second arm, models were trained solely on the In-House Clinical dataset and subsequently assessed on the BOT dataset’s validation set.

The results of this cross-dataset validation are summarized in **Table 10**. As anticipated, both models exhibited a degradation in performance when evaluated on previously unseen data. When trained on the BOT dataset and tested on the In-House Clinical dataset, the mAP of FC-YOLO decreased from 85.7% to 80.8% (a 4.9% drop), while the baseline YOLOv11s saw its mAP fall from 82.9% to 77.6% (a 5.3% drop). In the reciprocal scenario, the mAPs for FC-YOLO and YOLOv11s declined by 5.5% and 5.8%, respectively. This performance gap can be attributed to the domain shift between the datasets, likely arising from variations in staining protocols and histomorphological features. Crucially, however, FC-YOLO consistently outperformed YOLOv11s in terms of mAP across both cross-dataset scenarios, which more comprehensively reflects the effectiveness of our enhancements. Furthermore, the magnitude of performance degradation for FC-YOLO was smaller than that of the baseline model, indicating that the features learned by FC-YOLO are more generalizable. In summary, these internal-external cross-validation experiments confirm that, compared to the baseline YOLOv11s, FC-YOLO demonstrates superior generalization capability and robustness for the detection of gastric adenocarcinoma in pathological images.

**Table 10:** Cross-dataset validation performance comparison.

Training dataset	Validating dataset	Model	mAP (%)	Performance drop(%)
BOT dataset	In-House Clinical dataset	FC-YOLO	80.8	4.9
		YOLOv11s	77.6	5.3
In-House Clinical dataset	BOT dataset	FC-YOLO	77.3	5.5
		YOLO11s	74.4	5.8

### 3.8 Analysis of representative failure cases

A critical evaluation of any diagnostic AI model requires a thorough analysis of its failure cases. To better understand the limitations of FC-YOLO and contextualize its performance for potential clinical application, we examined representative false-positive (FP) and false-negative (FN) cases from the test set. This analysis provides valuable insights into which specific histopathological types of gastric cancer challenge the model’s detection capabilities and helps inform targeted strategies for future refinement.

**Figure 12:**
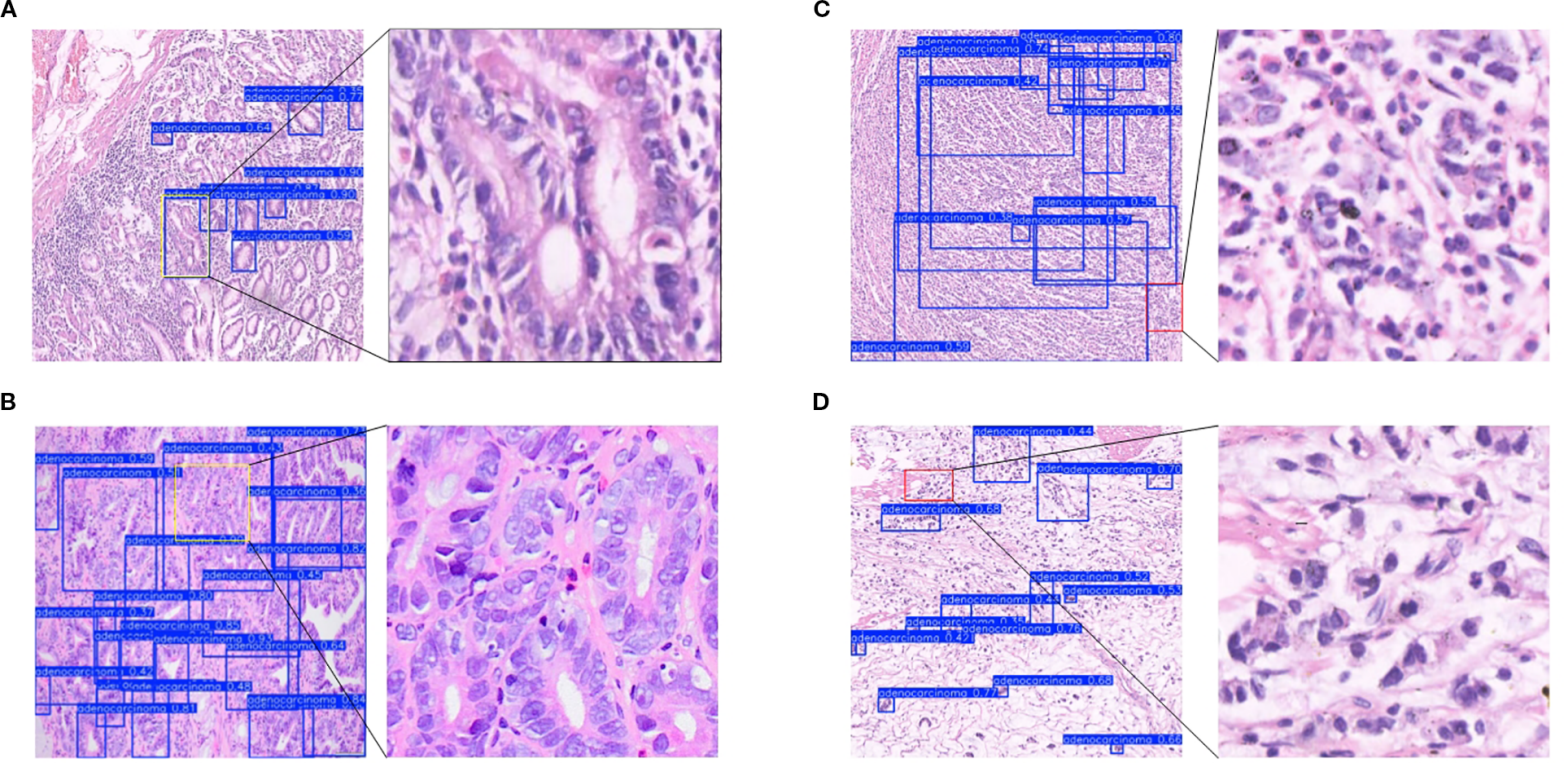
Analysis of misclassified cases. **(A)** Atypical hyperplasia. **(B)** High-grade intraepithelial neoplasia. **(C)** Poorly differentiated carcinoma. **(D)** Signet ring cell carcinoma.

Our analysis revealed that false positives (FPs) in both the BOT and In-House Clinical datasets occurred predominantly in regions of complex histological morphology with atypical hyperplasia (**Figure 12A**). These hyperplastic lesions often present with enlarged nuclei, hyperchromasia, pleomorphism, and prominent nucleoli—characteristics that closely resemble well-differentiated adenocarcinoma. Our model likely captured these high-grade cytological features, leading to the erroneous classification of the region as malignant. This type of error highlights a key limitation: the model struggles to distinguish between true neoplastic changes and the severe atypia found in benign reactive lesions. Another common source of FPs was high-grade intraepithelial neoplasia (HGIN) (**Figure 12B**). Morphologically, HGIN cells are nearly indistinguishable from carcinoma cells. The gold standard for differentiating HGIN from invasive carcinoma is the integrity of the basement membrane—specifically, whether it has been breached. As an object detection model, FC-YOLO is designed to identify overall cellular morphology and architecture, not to discern the integrity of a fine linear structure like the basement membrane. This task lies beyond the inherent capabilities of our object detection framework. Therefore, while the model’s classification of HGIN as malignant is clinically incorrect, it is a logically consistent outcome from a pattern recognition standpoint.

False negatives (FNs) represent a significant clinical risk. Our analysis revealed that FNs typically occurred in cases where malignant cells were sparsely distributed, poorly differentiated, or lacked clear architectural patterns. For instance (**Figures 12C, D**), poorly differentiated cancer cells often infiltrated the stroma loosely, either as single cells or in thin cords, with minimal to no formation of glandular structures. Another common source of FNs was signet ring cell carcinoma, a specific subtype of poorly differentiated adenocarcinoma. In these cases, the cytoplasm is filled with mucin, displacing the nucleus eccentrically to create a characteristic ‘ring-like’ appearance. These cells are frequently scattered individually and can be quite inconspicuous. The YOLO model, by its nature, learns to recognize targets based on their ‘shape’ and ‘structure’. When malignant cells do not form well-defined structures, such as the glandular tubes typical of more differentiated adenocarcinoma, the model may lack the distinct features required for confident identification.

In summary, our model faces two primary challenges: first, improving its ability to discriminate between malignant and benign structures amid atypical hyperplasia, and second, increasing its sensitivity to poorly differentiated and diffusely infiltrative cancers. This analysis directly informs our future research. We plan to augment our training dataset with more of these challenging cases, particularly signet ring cell carcinomas and high-grade intraepithelial neoplasia. Furthermore, incorporating subtype-specific annotations during the labeling process will be crucial, as this will enable the model to learn a richer repertoire of pathological features and ultimately enhance its overall discriminative power.

## 4 Discussion

This paper introduces FC-YOLO, a deep learning framework for the automated detection of gastric adenocarcinoma in histopathological images. The architecture is specifically designed to address key challenges in this domain, including complex backgrounds, high-density lesions, irregular glandular morphologies, and low feature contrast between malignant and benign regions. The FasterNet backbone enhances the model’s multi-scale feature representation while minimizing information loss during extraction. The MLCA attention mechanism improves detection accuracy by efficiently recalibrating channel-spatial features, all without increasing computational overhead. Furthermore, the CARAFE upsampling module replaces conventional methods to reduce model parameters and computational load, thereby accelerating inference speed. This synergistic design simultaneously improves both diagnostic precision and operational efficiency, making it well-suited for clinical histopathology applications.

It is noteworthy that our comparative experiments revealed a noteworthy phenomenon: for the specific task of detecting gastric adenocarcinoma, YOLOv5s marginally outperformed its more complex, larger-parameter counterparts, YOLOv7 and YOLOv8s. We hypothesize that this counterintuitive outcome stems from the dataset’s unique characteristics. Architectures like YOLOv7 and YOLOv8s are highly optimized for large-scale, general-purpose datasets such as COCO, but this performance does not always translate to specialized domains like medical imaging. Histopathological images differ significantly from general datasets, as they feature complex tissue backgrounds and high inter-class similarity. In this context, the relative architectural simplicity of YOLOv5s may be more effective at extracting salient features from pathological images. This finding underscores the principle that the most complex model is not invariably the optimal choice for a given application, highlighting the indispensable need for empirical validation tailored to the task at hand, rather than an overreliance on model complexity (42).

Furthermore, our results also revealed the promising potential of the RT-DETR model in this detection task, which, to some extent, underscores the advantage of Transformer architectures in capturing global contextual information and offers valuable insights for the future direction of medical pathology image recognition. However, our observations during training indicate that Transformer-based models generally entail a larger parameter count and greater computational complexity compared to purely CNN-structured YOLO models. Although RT-DETR optimizes efficiency by integrating a hybrid CNN backbone with partial Transformer layers, its training and inference phases still demand more substantial computational resources and prolonged durations relative to certain lightweight YOLO variants. Crucially, the YOLO series of models offers a significant advantage in terms of ease of engineering and deployment, making them more accommodating for hospitals with limited hardware resources. Therefore, from a comprehensive standpoint that considers detection accuracy, computational efficiency, and practical deployment feasibility, our proposed FC-YOLO model still demonstrates a superior overall advantage compared to the Transformer-integrated RT-DETR model for the task of gastric adenocarcinoma pathology detection.

This study innovatively adopts YOLOv11s as the foundational architecture for gastric adenocarcinoma detection in histopathological images, representing the first application of this framework in pathological image analysis. We selected a YOLO-series model due to its advantages for clinical deployment, including simplified implementation and real-time detection capabilities. The framework’s direct output of bounding box coordinates, confidence scores, and class probabilities enables seamless mapping to digital pathology coordinate systems. Furthermore, YOLO’s single-stage detection paradigm allows for immediate lesion localization in a single forward pass-a critical feature allowing pathologists to prioritize suspicious regions and minimize non-diagnostic viewing time. While YOLOv11s inherently offers favorable accuracy-speed balance, our FC-YOLO enhancements further optimize this equilibrium by improving detection precision while significantly reducing computational overhead, maintaining lightweight characteristics essential for clinical integration. The clinical applicability of YOLO-series architectures in pathological image analysis is well-established through precedent studies. Yu et al. (43) demonstrated YOLOv5s’ efficacy in mitotic figure detection within uterine smooth muscle tumors on whole slide images. Lee et al. (44) employed YOLOX to detect foci of lymphovascular invasion in gastric cancer and subsequently constructed an integrated deep learning model in conjunction with ConViT. Li et al. (45) enhanced YOLOv7 with BiFormer attention mechanisms, CARAFE upsampling, and GSConv modules, achieving improved accuracy and efficiency in vascular structure detection. Our experimental results corroborate CARAFE effectiveness in accelerating inference speed for histopathological analysis. The FC-YOLO framework demonstrates exceptional diagnostic performance with 82.8% mAP and 79.8% F1-score in gastric adenocarcinoma detection, particularly excelling in challenging scenarios involving complex stromal backgrounds and irregular neoplastic morphologies. This advancement holds significant clinical value by reducing diagnostic oversights and enhancing pathologists’ workflow efficiency through prioritized lesion localization.

Although our proposed model performs well, this study has several limitations. First, the model’s scope is confined to binary tumor versus non-tumor classification. It cannot perform more granular tasks, such as differentiating histological subtypes of adenocarcinoma (e.g., tubular, signet ring cell) or distinguishing adenocarcinoma from other malignancies like lymphoma or gastrointestinal stromal tumors. This specificity constrains its ability to provide comprehensive diagnostic support and limits its direct clinical applicability. Second, the study relies on a single-institution dataset, which restricts the model’s generalization capabilities. A lack of multi-center, multi-device validation, as well as validation on authoritative public datasets, may compromise its performance in real-world clinical settings. Finally, while we used heatmaps for model interpretability, our analysis lacks a quantitative metric, such as the Dice coefficient, to rigorously evaluate the concordance between the model’s attention and the true pathological regions.

Our future work will focus on addressing the following areas: enhancing the model’s generalizability by collecting data from multiple medical centers; optimizing and training the model to identify various cancer subtypes for multi-cancer classification, with a strong emphasis on clinical utility; and exploring deployment strategies on hospital-grade equipment to construct a pathology diagnostic system applicable to large-scale screening, rapid preliminary triage, and multi-target correlational analysis. A critical subsequent step will be the seamless integration of this system with existing digital pathology workflows. This will necessitate the development of compatible interfaces or plugins for whole-slide image (WSI) viewers such as QuPath and Aperio ImageScope. Upon loading a WSI into these platforms, our model would operate in the background, automatically identifying and displaying suspicious regions of gastric adenocarcinoma as either heatmap overlays or interactive bounding boxes. This functionality would enable pathologists to rapidly locate areas of interest, while the heatmap overlay format would serve to increase their confidence in the model’s findings. This approach does not render a diagnosis itself but rather flags regions that warrant expert review, thereby reducing the risk of missing minute or atypical foci, particularly under heavy workloads. In essence, the system is not intended to replace pathologists but to serve as an auxiliary tool that assists them in rapidly locating suspicious regions and provides diagnostic support, thus alleviating their workload. The ultimate objective is for the FC-YOLO technology to deliver tangible benefits to the patient population.

## 5 Conclusion

In this research, we propose an object detection model designated FC-YOLO, which employs deep learning techniques for the automated detection of gastric adenocarcinoma in pathological images. We replaced the backbone of YOLOv11s with FasterNet and integrated the MLCA attention mechanism and the CARAFE upsampling module. These modifications enhance the extraction of multi-scale critical features, thereby improving both the model’s detection accuracy and inference speed. Experimental results obtained from the BOT dataset and a dataset collected from hospital sources demonstrate that FC-YOLO outperforms current mainstream object detection models in identifying gastric adenocarcinoma within pathological images, particularly in scenarios with complex backgrounds and for small target lesions. Our future research will pursue several avenues: (i) collecting a more extensive and varied pathological image dataset, encompassing a broader range of cancer categories and lesion types; (ii) To develop a lightweight pathology diagnostic system based on our model for deployment on devices in real-world clinical settings, thereby providing diagnostic assistance to physicians. (iii) further optimizing computational efficiency through methods such as model pruning and knowledge distillation.

## Data Availability

Publicly available datasets were analyzed in this study. This data can be found at Zenodo: https://zenodo.org/records/15129785. The private dataset is not available because of privacy regulations. The trained models for this project are available at https://github.com/BTMC-JPAI/FC-YOLO.
